# Foreword to the special issue on advances in neuroendocrine neoplasms

**DOI:** 10.1007/s11154-021-09639-z

**Published:** 2021-03-12

**Authors:** Giovanni Vitale, Antongiulio Faggiano

**Affiliations:** 1 Laboratory of Geriatric and Oncologic Neuroendocrinology Research, Istituto Auxologico Italiano, IRCCS, Cusano Milanino (MI), Milan, Italy; 2 Department of Medical Biotechnologies and Translational Medicine, University of Milan, Milan, Italy; 3 Endocrinology Unit, Department of Clinical and Molecular Medicine, Sant’Andrea Hospital, Sapienza University of Rome, Rome, Italy

Neuroendocrine neoplasms (NENs) are a heterogeneous and broad class of tumors originating from neuroendocrine cells. These cells are widely dispersed all over the human body with different genetic and histological characteristics [1, 2]. Therefore, these tumors occur almost everywhere in the body and display a highly variable prognosis. NENs can cause a wide array of symptoms and complications depending on the type of tumor, its location and the production of several factors. These factors can seriously compromise organs homeostasis and quality of life of patients [3–6].

All these levels of heterogeneity, together with the rarity of some NENs, drastically hampers the possibility of implementing high-scale translational and clinical studies. In this scenario, animal models that faithfully recapitulate clinical features and related complexity of NENs are needed in preclinical research [7, 8]. However, several therapeutic strategies and drugs are available in the complex management of NENs [9, 10].

This special issue aims to illustrate how much has been achieved and what remains to be done with regard to NENs. This collection covers some important new topics, but also shows how management has evolved and different approaches have been introduced in patients with this complex and heterogenous disease.

The somatostatin system plays an important role in the pathogenesis, diagnosis and treatment of NENs. Klomp et al. reveal the secrets of epigenetics in the modulation of somatostatin receptors, opening a new scenario in the diagnosis and treatment of these tumors through the possibility to target the epigenetic machinery.

In the last years there is mounting evidence supporting the role of the gut microbiome in the pathogenesis of several tumors and response to the therapy. Vitale et al. provide an overview concerning the complex interplay between gut microbiota and gastroenteropancreatic NENs, focusing on the potential role in tumorigenesis and progression in these tumors.

La Rosa and Uccella discuss the lights and shadows of the current WHO classifications used to define and characterize NENs of the pituitary gland, lung, breast and those of the head and neck region, and digestive and urogenital systems.

Pirasteh et al. provide a pictorial review of the different imaging techniques and their role and utility in management of patients with NENs, aimed to provide a practical guide for all clinicians.

Oleinikov et al. describe novel insights in the pathophysiology, diagnosis and treatment of carcinoid heart disease.

Theranostics in neuroendocrine tumors (NETs) is actually based on somatostatin analogues radiolabeled with β-emitters radionuclides, but it is open to several and fascinating new scenarios. Albertelli et al. analyze the main predictive clinico-pathologic factors of response to peptide receptor radionuclide therapy, which is now the main therapeutic approach for G1-G2 gastro-entero-pancreatic NETs progressing under therapy with somatostatin analogues. Refardt et al. provide an overview on present and future applications of theranostics in patients with NETs.

Espinosa-Olarte et al. critically review available evidence for the use of chemotherapy in lung and gastroenteropancreatic NENs, focusing on its current role in the treatment algorithm of this tumors.

In addition, this issue covers the current evidence and future perspectives of immunotherapy in NENs.

Vuillerme et al. assess the role of RECIST criteria in the evaluation of response to therapy in metastatic NENs. Tumor size variation represents a central parameter for tumor response assessment, but others need to be investigated.

White et al. evaluate cost-effectiveness in the management of NENs. This approach is of great relevance in all cancers but still more in NENs, due to the long-time survival of this patients and the wide use of costly therapies.

In summary, this special issue is a helpful tool for clinicians, by providing updated reports on diagnosis and therapy of NENs, as well as for researchers, by suggesting different areas of technological and cultural improvements and further investigation in the field of NENs.

## Abbreviations

NENs: Neuroendocrine neoplasms
NETs: Neuroendocrine tumors

## References

[1] Pedraza-Arévalo S, Gahete MD, Alors-Pérez E, Luque RM, Castaño JP (2018). Multilayered heterogeneity as an intrinsic hallmark of neuroendocrine tumors. Rev Endocr Metab Disord.

[2] Walenkamp A, Crespo G, Fierro Maya F, Fossmark R, Igaz P, Rinke A (2014). Hallmarks of gastrointestinal neuroendocrine tumours: implications for treatment. Endocr Relat Cancer.

[3] Tamagno G, Bennett A, Ivanovski I (2020). Lights and darks of neuroendocrine tumors of the appendix. Minerva Endocrinol.

[4] Fanciulli G, Ruggeri RM, Grossrubatscher E, Calzo FL, Wood TD, Faggiano A (2020). Serotonin pathway in carcinoid syndrome: Clinical, diagnostic, prognostic and therapeutic implications. Rev Endocr Metab Disord.

[5] Fuentes-Fayos AC, García-Martínez A, Herrera-Martínez AD, Jiménez-Vacas JM, Vázquez-Borrego MC, Castaño JP (2019). Molecular determinants of the response to medical treatment of growth hormone secreting pituitary neuroendocrine tumors. Minerva Endocrinol.

[6] Alexandraki KI, Tsoli M, Kyriakopoulos G, Angelousi A, Nikolopoulos G, Kolomodi D (2019). Current concepts in the diagnosis and management of neuroendocrine neoplasms of unknown primary origin. Minerva Endocrinol.

[7] Carra S, Gaudenzi G (2020). New perspectives in neuroendocrine neoplasms research from tumor xenografts in zebrafish embryos. Minerva Endocrinol.

[8] Gaudenzi G, Albertelli M, Dicitore A, Würth R, Gatto F, Barbieri F (2017). Patient-derived xenograft in zebrafish embryos: a new platform for translational research in neuroendocrine tumors. Endocrine.

[9] Vitale G, Dicitore A, Sciammarella C, Di Molfetta S, Rubino M, Faggiano A (2018). Pasireotide in the treatment of neuroendocrine tumors: a review of the literature. Endocr Relat Cancer.

[10] Rindi G, Wiedenmann B (2020). Neuroendocrine neoplasia of the gastrointestinal tract revisited: towards precision medicine. Nat Rev Endocrinol.

